# Electronic Property and Negative Thermal Expansion Behavior of Si_136-*x*_Ge*_x_* (*x* = 8, 32, 40, 104) Clathrate Solid Solution from First Principles

**DOI:** 10.3390/nano9060851

**Published:** 2019-06-03

**Authors:** Dong Xue, Charles W. Myles

**Affiliations:** Department of Physics and Astronomy, Texas Tech University, Lubbock, TX 79409-1051, USA; charley.myles@gmail.com

**Keywords:** electronic density of states, phonon anharmonicity, macroscopic Grüneisen parameter, free energy, vibrational entropy

## Abstract

We present the electronic and vibrational studies on Si_136-*x*_Ge*_x_* (*x* = 8, 32, 40, 104) alloys, using the local density approximation (LDA) scheme. We find that a “nearly-direct” band gap exists in the band structure of Si_104_Ge_32_ and Si_96_Ge_40_, when compared with the similarly reported results obtained using a different computational code. The calculated electronic density of state (EDOS) profiles for the valence band remain nearly identical and independent of the Ge concentration (*x =* 32, 40, 104) even though some variation is found in the lower conduction band (tail part) as composition *x* is tuned from 8 (or 40) to 104. The negative thermal expansion (NTE) phenomenon is explored using quasi-harmonic approximation (QHA), which takes the volume dependence of the vibrational mode frequencies into consideration, while neglecting the temperature effect on phonon anharmonicity. Determined macroscopic Grüneisen parameter trends show negative values in the low temperature regime (1 K < *T* < 115 K), indicating the NTE behavior found in Si_128_Ge_8_ is analogous to the experimental result for Si_136_. Meanwhile, calculations for the ratio of the vibrational entropy change to the volume change at several characteristic temperatures reconfirm the existence of NTE in Si_128_Ge_8_ and Si_104_Ge_32_.

## 1. Introduction

Empty alloy clathrates are a class of materials with either a crystalline or an amorphous framework comprising more than one Group IV element (e.g., Si, Ge, and Sn). These open-framework compounds are constructed from bonding configurations and exhibit *sp*^3^ hybridized geometry. The two primary structures of these materials are similar to Type-I and Type-II clathrate hydrates [1] for which the unit cells are coordinated by polyhedral cavities that can encapsulate guest atoms. Specifically, there are 46 atoms in a Type-I primitive unit cell and 136 framework atoms in a Type-II enlarged unit cell that consists of the building blocks of the 28-atom and 20-atom (or 24-atom) polyhedra. The present work focuses on the Type-II Si_136-*x*_Ge*_x_* (0 < *x* < 136) SiGe solid solution alloy comprising 20-atom (dodecahedron) cages and 28-atom (hexakeidacahedron) cages connected in the ratio of 4:2. Some understanding of the Type-II SiGe clathrate solid solution has been obtained by experimental and theoretical studies during the past two decades. Specifically, in previous work, Baranowski et al. classified the phase structures of the synthesized Si_136-*x*_Ge*_x_* into two categories based on the synthesized Ge content *x* as follows [2]. The stoichiometric amount (*x*) of Ge for amorphous formation ranges from approximately 20.4 to 68, while the concentration values corresponding to crystalline Si_136-*x*_Ge*_x_* satisfy 0 < *x* < 20.4 and 68 < *x* < 136. Our theoretical work on electronic and vibrational properties of Si_136-*x*_Ge*_x_* is based on the assumption that all of these alloy clathrates with the assigned composition are found in the crystalline phase. Using the Cambridge sequential total energy package (CASTEP) package [3], K. Moriguchi et al. investigated the electronic properties of Si_136-*x*_Ge*_x_*, for which the nearly-direct and direct wide band gaps were found to range from 1.2 to 2.0 eV for *x* = 8, 32, 40, 96, 104, and 128 [4]. The electronic density of state profiles that were calculated using the Vienna ab initio simulation package (VASP) code show the existence of band gaps with values that are compared with the results of Koji et al. for *x* = 8, 40, and 104.

Recently, anomalous negative thermal expansion (NTE) behavior in silicon-based clathrate has attracted increasing research interest [5,6]. For example, Tang et al. investigated the thermal properties of Si_136_ and reported an NTE region exists between in the 10–140 K temperature range in such a pure framework [7]. Negative thermal expansion is defined as a phenomenon where the material contracts rather than expands with an increase in the temperature. Prior to the work of Tang et al. on pure Si_136_, NTE in diamond phase alloy Si_1-*x*_Ge*_x_* was discussed for *x* = 0 [8,9,10,11,12], while Si_1-*x*_Ge*_x_* (*x* = 1) was found to demonstrate relatively weak NTE for temperatures lower than approximately 40 K [13]. Thus, the motivation for much of our work has been the first-principles prediction of NTE behavior appearing in the Si_136-*x*_Ge_*x*_ clathrate alloy with silicon as the dominant component. In this paper, we mainly report the results for the volume(*V*)-dependent vibrational entropy and free energy of pure Si_136_, and the results for the dispersion relationships and macroscopic Grüneisen parameters of Si_136-*x*_Ge*_x_* (*x* = 0, 8, 32). 

To the best of our knowledge, while some computational studies have been reported in the literature [14,15,16,17,18] on the vibrational properties of Type-I and Type-II clathrate materials using density-functional-theory-based techniques within the harmonic approximation (HA), there has been no discussion of the anharmonic effects associated with clathrate materials’ lattice dynamics. To effectively explore anharmonic properties such as the NTE behavior found in Si_136-*x*_Ge_*x*_, we present two approaches for probing this anomalous thermal performance that rely on the quasi-harmonic approximation (QHA) formalism [19]. Essentially, the core of the QHA scheme lies solely in the volume-dependence of the phonon anharmonicity, whereas the temperature is assumed to remain independent of the phonon dispersion spectrum. It is well-known that the VASP code always evaluates the lattice (phonon) dispersion spectrum by determining the force-constant matrix ***D***(***q***) within the HA scheme and at *T* = 0. On the basis of the thus-derived phonon mode frequency *ω*_j_(***q***) derived from ***D***(***q***), the predicted NTE behavior is mathematically manifested by the negative thermal expansion coefficient that corresponds to the negative weighted average of the mode Grüneisen parameter *γ*_j_ (defined as –In*ω*_j_(***q***)/In*V*) in the certain temperature range. The subscript *j* here denotes the phonon branch.

In addition to the macroscopic Grüneisen parameter *γ*(*T*) obtained from the weighted average of *γ*_j_, the volume derivative of the vibrational entropy *S*_vib_ is another thermodynamic quantity used for examining NTE behavior. This approach for predicting NTE is effective due to two factors. First, the NTE coefficient scales linearly with the volume derivative of the vibrational entropy (written as ə*S*_vib_/ə*V*). The second reason is that the Si_136-*x*_Ge*_x_* system is supposed to be a perfect crystalline lattice, where the temperature-dependent contribution to the entropy arising from lattice vibrations is considered. Meanwhile, other types of anharmonic effects and electronic and magnetic contributions are assumed to be too small and are neglected. This alternative approach for exploring NTE is still within the QHA framework, because the ə*S*_vib_/ə*V* term contains the information regarding the volume-dependence of the HA-based phonon frequency *ω*_j_(***q***) appearing in the vibrational entropy. In this work, both of these two approaches were applied to verify that the Si-dominated Si_128_Ge_8_ (or Si_104_Ge_32_) alloy demonstrates the NTE effect at a temperature range that is comparable to that of pure Si_136_.

## 2. Computational Approach

We present the results of first-principles density-functional-theory (DFT)-based study on the electronic and vibrational properties of clathrate solid solution Si_136-*x*_Ge*_x_* (0 ≤ *x* < 136) and chose to use the VASP code [20]. We employed the Ceperley–Alder exchange-correlation potential along with the pseudopotentials obtained using the projector augmented wave (PAW) method. All of the calculations reported here are based on local density approximation (LDA) in which the self-consistent Kohn–Sham equations [21] are solved. This implementation has been extensively and successfully examined in a wide range of material systems [22,23,24] and appears to be highly efficient for the large clathrate unit cells involved in this work, namely, the Si- and Ge-containing Type-I, II, VIII compounds [25,26,27]. The energy cutoff parameter was set to be 300 eV for Si_136-*x*_Ge*_x_*, in the context of performing electronic property calculation. 

In all cases, geometry optimization is the initial task of our computations and is performed after selecting a fixed lattice constant and relaxing the internal coordinates by means of the conjugate gradient (CG) algorithm. Next, several pairs of the obtained data describing the LDA energy vs. volume were fitted to the third-order Birch–Murnaghan equation of state (EOS) [28], obtaining an energy–volume relationship. Moreover, on the basis of this fitting procedure, equilibrium state parameters including the minimum binding energy and lattice parameter can be determined. We perform Brillouin zone integration using a 4 × 4 × 4 Monkhorst-Pack *k*-point grid [29] in order to perform the relaxation and ultimately to characterize the equilibrium geometry. The total energy convergence criterion was set to 10^−7^ eV. In this paper, we discuss the electronic band structure (BS) with respect to Si_128_Ge_8_ and evaluate the density of states at the minimized energy configurations of Si_136-*x*_Ge*_x_* (*x* = 8, 40, 104).

To investigate the lattice dynamics of these SiGe clathrate solid solution compounds, 2 × 2 × 2 Monkhorst-Pack *k*-point sampling was applied to obtain the Γ-point vibration modes and the dispersive relationships were derived from the dynamical matrix ***D***(***q***). Here, ***q*** acts as the phonon wave vector in the first Brillouin zone (BZ). The determination of ***D***(***q***) was carried out in two steps. The first step was to obtain the 3*N* × 3*N* matrix in terms of the exact HA for ***q*** = (0,0,0) (Γ-point), where *N* denotes the total number of atoms in the optimized primitive unit cell. To do this, each atom in the polyhedron cage is moved by a small finite displacement *U*_0_ (= 0.02 Å) from equilibrium. The second step is to approximately obtain the 3*N* × 3*N* matrix ***D***(***q***) (***q*** ≠ (0,0,0)) for ***q*** confined within the vicinity of Γ-point. The details of this computational procedure were reported in a previous paper [30]. After collecting the entire ***D***(***q***), we diagonalize this matrix and determine the vibrational mode eigenvalues *ω*_j_^2^(***q***) (squared frequencies) and the eigenvectors.

In the same HA, we calculated the vibrational entropy from the vibrational free energy which is originally determined from the vibrational mode frequencies *ω*_j_^2^(***q***). Using the quasi-harmonic approximation, the volume dependence of the vibrational entropy at various characteristic temperatures was evaluated. Similarly, another thermodynamic property describing phonon anharmonicity was obtained from the inspection of the volume dependence of the vibrational frequencies *ω*_j_(***q***). Specifically, the original definition of microscopic Grüneisen parameter *γ*_i_(***q***) = –In*ω*_j_(***q***)/In*V* can be approximated as the negative ratio of the fractional change in the mode frequency Δ*ω*_j_(***q***)/*ω*_j_(***q***) to the fractional change in volume Δ*V*/*V* using the Feynman–Hellmann theorem [31] based on the finite difference method (FDM). To do this, phonon anharmonicity calculations for determining *γ*_i_(***q***) were repeated at three volume points consisting of one equilibrium volume and two additional volume values that are slightly larger and smaller, respectively. Moreover, the volume derivative of the dynamical matrix elements (*D_ij_*(***q***)) was approximated as Δ*D_ij_*(***q***)/Δ*V*.

## 3. Results and Discussion

### 3.1. Electronic Properties

**Figure 1:**
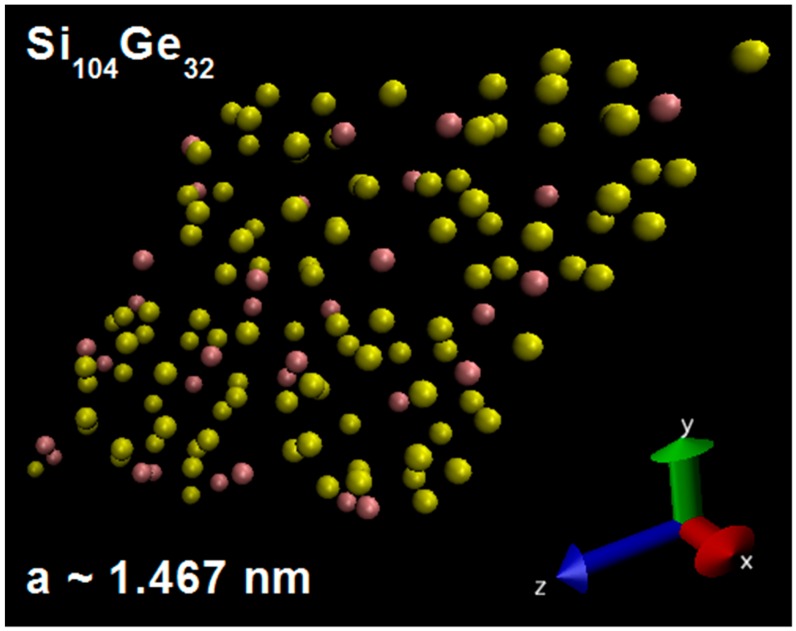
Cubic unit cell of Si_104_Ge_32_. The yellow solid balls denote the Si atoms while the purple solid ones represent the Ge atoms that act as substitutional framework hosts.

Before discussing the exact results of electronic and vibrational properties, it should be mentioned that the SiGe alloy models used in this work were Si_128_Ge_8_, Si_104_Ge_32_, Si_96_Ge_40_, and Si_32_Ge_104_, assuming full occupation on the 8*a*, 32*e*, 8*a* + 32*e*, and 8*a* + 96*g* Wyckoff sites by Ge. It is necessary to display the microscopic structure of Si_104_Ge_32_ which is based on the 136-atom unit cell. As is shown in Figure 1, our determined lattice constant *a* is about equal to 1.467 nm which lies in the size range of 1 to 1000 nanometers, satisfactory with the definition of nanomaterial [32].

**Figure 2:**
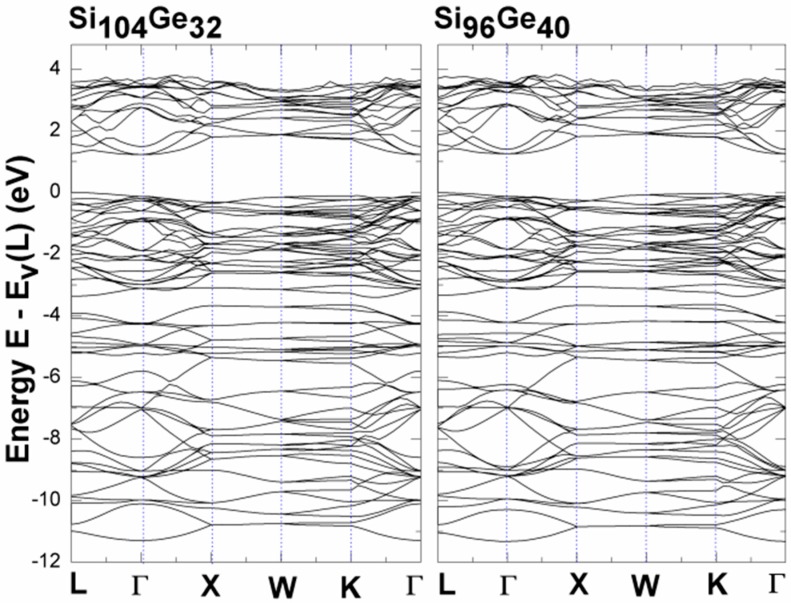
Local density approximation (LDA)-determined electronic band structures of Si_104_Ge_32_ and Si_96_Ge_40_, where the zero of energy is set to be the valence band maximum at the L point.

Many reports [33,34,35] have shown that some Si semiconductor clathrates have wider energy band gap than that of the pristine cubic diamond silicon (*cd*-Si) that has the band gap of 1.17 eV [36]. Based on these reports, the evaluation of the electronic band gap of Si_136-*x*_Ge*_x_* with the specified Ge concentration may provide guidance for the search for promising candidate materials for application in optoelectronic semiconductor devices. Figure 2 demonstrates the electronic band structure calculated by us using VASP for Si_128_Ge_8_ that has a structural symmetry specified by the Fd-3m space group [37]. It is clearly observed that the top of the valence band is found at the L high-symmetry point. The depicted band structure shown in the energy range of 0 and 2 eV enables Si_136-*x*_Ge*_x_* (*x* = 32, 40) to exhibit the so-called “nearly-direct” band gap because the eigenenergy of the conduction band edge at L is slightly higher than the eigenenergy of the conduction band edge at the Γ point. In other words, the degeneracy of the lowest conduction band at the L and Γ points is not noticeably different. Our predicted results for the band gap magnitude (~1.23 eV in Si_104_Ge_32_; ~1.27 eV in Si_96_Ge_40_) and “nearly-direct” behavior are compared with the first-principles result (~1.22 eV and ~1.25 eV) of Koji et al. [4] for Si_104_Ge_32_ and Si_96_Ge_40_, in which the CASTEP code was utilized to perform their calculations. 

**Figure 3:**
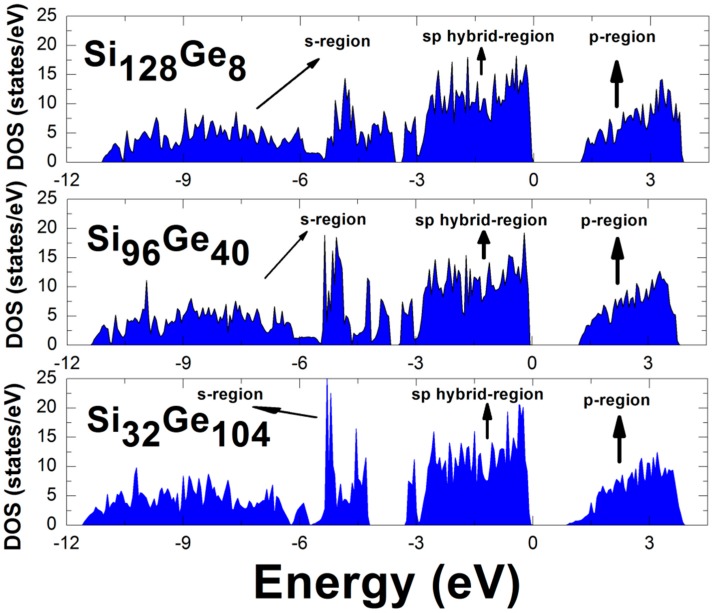
Calculated electronic density of states for Si_136-*x*_Ge*_x_* (*x* = 8, 40, 104), zero energy is set at the maximum of the valence band.

Ground state computation at *T* = 0 can facilitate a comprehensive study for determining the material’s electronic properties such as the LDA-derived electronic density of states (EDOS). Considering Ge-containing silicon clathrate Si_136-*x*_Ge*_x_* with the compositions *x* = 8, 40, 104, the empty framework of these alloys without encapsulated guest behaves structurally as the four-fold-coordinated sp^3^ configuration, while all of the valence electrons of Group IV atoms follow the Zintl–Klemm concept to form covalent bonds [38]. Accordingly, no charge transfer occurs in Si_136-*x*_Ge*_x_* (*x* = 8, 40, 104) because all valence electrons are used to build the charge-balanced composition, enabling the material to be a semiconductor and to play a promising role in thermoelectric applications. Figure 3 shows the results of our DFT calculations for examining the EDOS as a function of the specified Ge composition, assuming that three inequivalent sites (8*a*, 32*e*, 96*g*) of unit cell geometry are completely filled by the same Group IV element instead of the mixture of Si and Ge. An examination of Figure 3 shows that there are no major differences between EDOS profiles of Si_128_Ge_8_ and Si_96_Ge_40._ However, it is observed that the lower region of the conduction band in the EDOS profile of Ge-dominant alloy Si_32_Ge_104_ is “downshifted”, leading to a reduced optical band gap (~0.83 eV) in comparison with the other materials (1.27–1.39 eV). Additionally, the EDOS profile of Si_32_Ge_104_ for the conduction band region has a different tail region located between approximately 0.8 eV and 1.6 eV, indicating that the energy states surrounding the minimum energy (at approximately 1.15 eV) are becoming sparsely populated. Generally, the electronic densities of states of Si_136-*x*_Ge*_x_* (*x* = 8, 40, 104) are sensitive to the apparent change in the Ge concentration. Specifically, the change from the Si-dominant Si_128_Ge_8_ alloy, to Si_96_Ge_40_ and then to the Ge-dominant Si_32_Ge_104_ alloy is accompanied by a variation in the conduction band profile occurring at the lower region and a reduction in the optical band gap.

**Figure 4:**
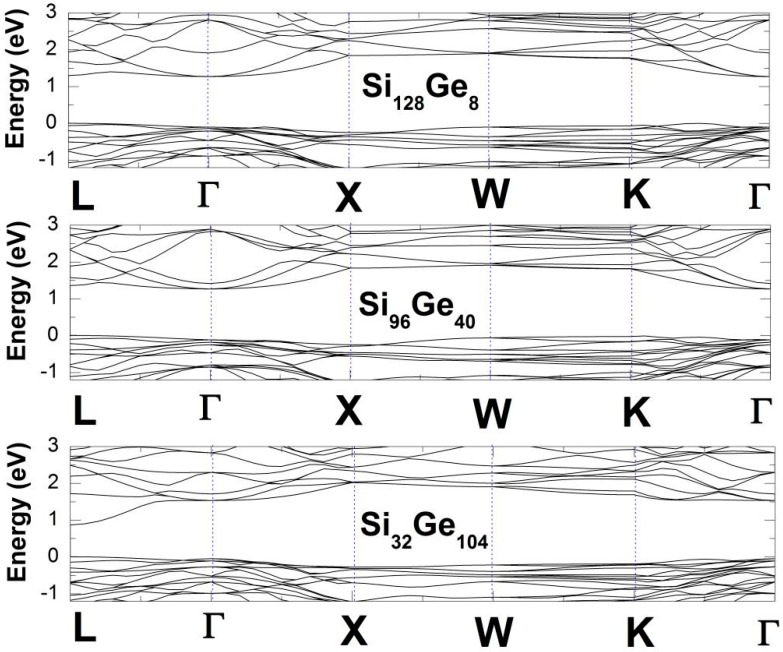
Electronic band structures of Si_136-*x*_Ge*_x_* (*x* = 8, 40, 104).

It should be mentioned that, the LDA band structure calculation underestimates the band gap [39]. We see that, adding substitutional Ge atoms (from 8 to 40 to 104) to the framework slightly modifies the band structure (see Figure 4). Increasing the substitutional Ge atoms modifies several states near the valence band maxima and conduction band minima, thus reducing the band gap. In all of the below structures, the smallest energy gap lies along the L to the Γ line. We consequently conclude that these compounds have “nearly-direct” band gaps because the eigenenergy at the L point is always slightly higher than that of the Γ point. In addition to these, each electronic density of states (EDOS) in Figure 3 shows three major regions, which can be connected to an s-region, an sp hybrid-region and a p-region. It is known that, the appearance of the gap in the valence band region is due to the five-ring patterns of the Ge or Si atoms [4,40] but there still exists some criticism about this statement [4]. According to ref. [41] the tetrahedrally bonded framework atoms (Si and Ge) possess small angular distortion. It is impossible to express the valence band maximum on an absolute scale, because of the self-consistent plane wave calculation. To this end, the EDOS in Figure 3 are qualitatively very much similar to one another in the three alloy materials that we have investigated so far. 

### 3.2. NTE Behavior Investigation

**Figure 5:**
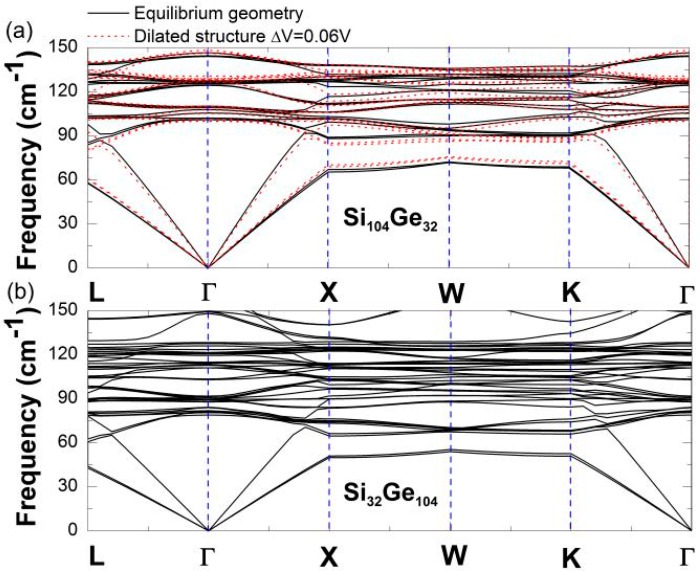
Low-frequency dispersion relationship curves of (**a**) Si_104_Ge_32_ and (**b**) Si_32_Ge_104_ in the Brillouin zone, for the optimized geometry (black solid line) and dilated configuration (red dotted line).

The lowest-lying acoustic mode regions are of greater importance than the other regions of the phonon spectrum due to the low-*T* anomalous negative thermal expansion. Consequently, we begin with the first-principles calculations (Figure 5) of the low-frequency (0–150 cm^−1^) dispersion spectrum of Si_136-*x*_Ge*_x_* (*x* = 32, 104) in the Brillouin zone. This calculated dispersion curve primarily displays the longitudinal acoustic (LA) phonon and transverse acoustic (TA(1) and TA(2)) phonons with double degeneracy along specific directions involving the Γ-L, Γ-X, and Γ-K lines. Meanwhile, Figure 5a shows how geometry dilation affects the vibrational spectrum of Si_104_Ge_32_ in which the fractional change in the mode frequencies of the TA(1) and TA(2) phonons increase with increasing volume (Δ*V*/*V* = +6%). By contrast, the frequency of the longitudinal phonon decreases upon structural dilation. For the low-frequency spectrum of optimized Si_32_Ge_104_, Figure 5b shows that the phonon velocity stays nearly unchanged. 

**Figure 6:**
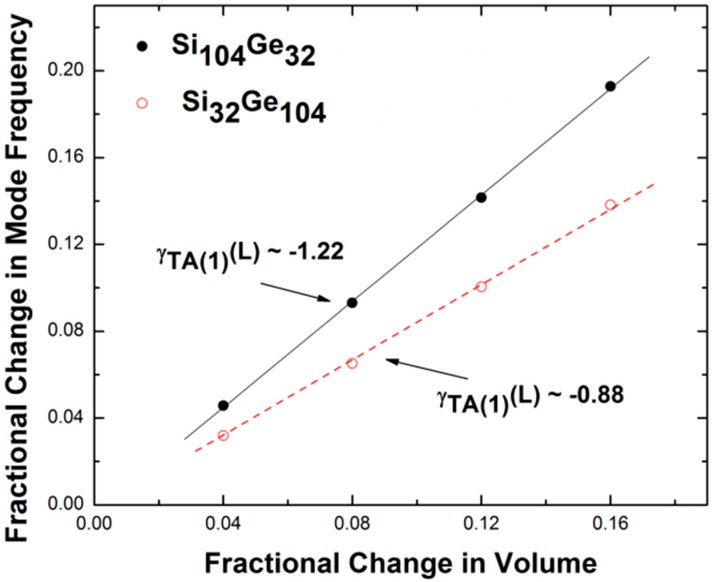
Variation in the fractional change in the mode frequency with respect to the TA(1) phonon branch along the Γ-L direction, as a function of the fractional change in the volume.

Figure 6 shows the quantized collective motion of the lattice framework atoms responses in Si_32_Ge_104_ and Si_104_Ge_32_ upon structural variation using the finite difference method in combination with the Feynman–Hellmann theorem: Δ*ω*_TA(1)_(L)/*ω*^0^_TA(1)_(L) = –*γ*_TA(1)_(L)Δ*V*/*V*, where *ω*^0^_TA(1)_(L) denotes the transverse acoustic phonon mode frequency at the L point at the optimized geometry without the structural change and Δ*ω*_TA(1)_(L) describes the change in such mode frequency. The fractional change in the equilibrium volume (Δ*V*/*V*) is evaluated as the absolute difference between the slightly larger volume and the slightly smaller volume and is equal to 0.04 *V*, 0.08 *V*, 0.12 *V,* and 0.16 *V*. Moreover, the fractional change in the mode frequency of the TA(1) phonons in Si_32_Ge_104_ increases more slowly with increasing Δ*V*/*V* in comparison with that of Si_104_Ge_32_. In the same figure, the solid and dashed lines act as a guide for the eye, demonstrating the constant positive slope for the ratio of Δ*ω*_TA(1)_(L)/*ω*^0^_TA(1)_(L) to Δ*V*/*V*. Multiplying this ratio by −1 defines the mode Grüneisen parameter of the TA(1) phonon localized at the L point (*γ*_TA(1)_(L)). Specifically, the determined ratio of *γ*_TA(1)_(L) for Si_32_Ge_104_ to *γ*_TA(1)_(L) for Si_104_Ge_32_ is approximately equal to 0.72, indicating that the lattice framework is accompanied by a weak vibrational response with geometry variation when the Ge content is dominant. 

Numerical calculations of the mode Grüneisen parameter (*γ*_j_) of the specific phonon mode using FDM are listed in Table 1. The thus-evaluated values are obtained at the X and Γ high-symmetry points of the dispersion curves in the [100] direction. Due to the existence of a diverging mode Grüneisen parameter at the Γ point, the results for the *γ*_j_(Γ) of the TA(1) and LA phonons are computed in the vicinity of (0,0,0). Table 1 also shows that the transverse acoustic phonons have *γ*_j_ values below zero. 

**Figure 7:**
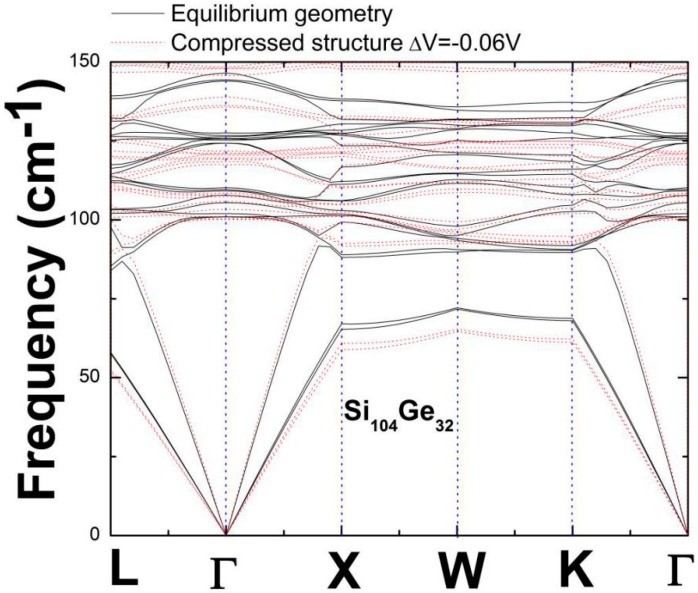
Low-frequency dispersion relationship curves of Si_104_Ge_32_ in the Brillouin zone, for the optimized geometry (black solid line) and compressed configuration (red dotted line).

To explore how anharmonic frequencies which are smaller than harmonic ones affect the sign of corresponding mode Grüneisen parameter in the case of Si_104_Ge_32_, we consider this time the comparison between the compressed structure (−6%V) and the optimized one, in the presence of dispersion relations. Figure 7 shows the mode frequency variation confined within the low-frequency (0–150 cm^−1^) regime, leading to smaller values of transverse acoustic phonon modes when paying attention to the compressed configuration that is numerically given by −0.06 *V*. These anharmonic frequencies still give rise to negative Gruneisen parameters according to Δ*ω*_TA_(***q***)/*ω*^0^_TA_(***q***) = –*γ*_TA_(***q***)Δ*V*/*V,* because at this moment Δ*V* becomes negative in combination with negative Δ*ω*_TA_(***q***). On the other hand, the lowest-lying longitudinal optic (LO) and transverse optic (TO) phonons only display positive Grüneisen parameters, while most of the other remaining optic phonons (lying above the acoustic bands) show positive sign with respect to *γ*_j_ according to our DFT-determination. 

The overall Grüneisen parameter *γ*(*T*) serves as a measure of anharmonicity of lattice vibrations, while playing an essential role in giving rise to NTE behavior. Our first-principles method utilizes the weighted average to obtain *γ*(*T*) using the equation *γ*(*T*) = ∑_j_ *γ*_j_*C*_V_/∑_j_ *C*_V,j_. where *C*_V,j_ is the partial vibrational mode contribution to the heat capacity originating from the phonon mode frequency, *ω*_j_(***q***) and its value always remains positive. Based on the data presented in Table 1, the listed transverse acoustic phonon modes have a larger negative contribution to the macroscopic Grüneisen parameter *γ*(*T*) while *T* is restricted to the low-temperature regime (below approximately 150 K). This is because *C*_V,j_ evaluated at the low-temperature region for the TA phonons is dominant compared to the remaining phonon contribution to the heat capacity. 

**Figure 8:**
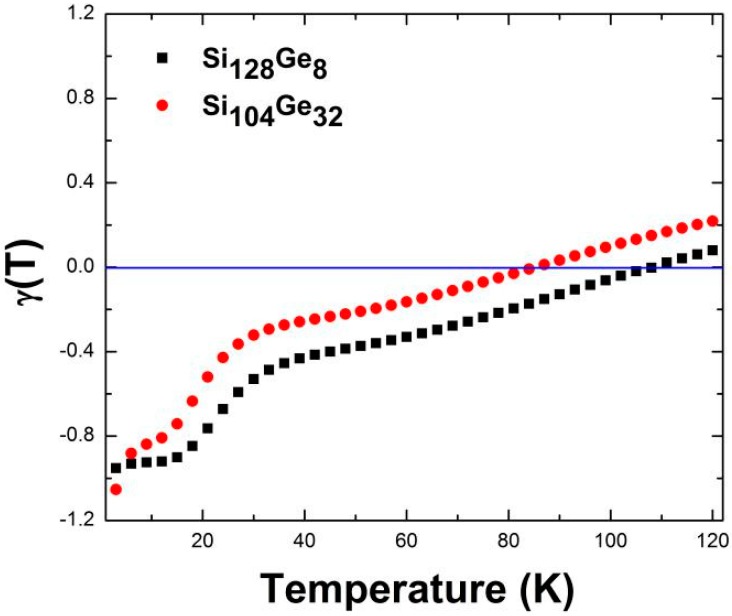
DFT-predicted macroscopic Grüneisen parameters of Si_128_Ge_8_ and Si_104_Ge_32_.

In addition, the thermodynamic relationship *α*_v_(*T*) = *γ*(*T*) *C*_V_*ρ/K*_T_ states that the sign of the volumetric thermal expansion coefficient *α*_v_(*T*) directly depends on the negative or positive sign of *γ*(*T*) because the bulk modulus at the specified temperature *K*_T_, and the heat capacity *C*_V_ along with material’s density *ρ* always remain positive. Briefly, negative thermal expansion is indicated by the negative sign of *γ*(*T*). Our calculated *γ*(*T*) profile for Si_136-*x*_Ge*_x_* (*x* = 8, 32) is presented in Figure 8, whereas *T* is limited to the range of 1–122 K. It is observed that the macroscopic Grüneisen parameters of Si_128_Ge_8_ are always negative in the 1–108 K range, while Si_104_Ge_32_ have similar temperature profiles showing negative *γ*(T) region spanning the range of 1–84 K. 

**Figure 9:**
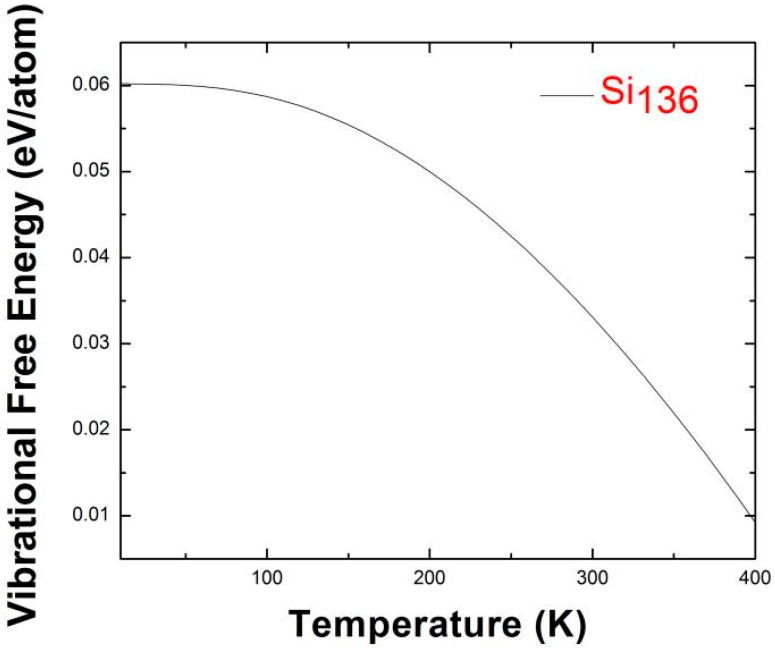
Predicted vibrational free energy including zero-point contribution for Si_136_, in the range of 10–400 K.

In the harmonic approximation, the vibrational contribution to the free energy is temperature-dependent and is given by *F*_vib_ = *k*_B_*T*∑**_q_**∑_j_In{2sinh(*hω*_j_(***q***)/4*πk*_B_*T*)}, where *k*_B_ is Boltzmann’s constant and *h* is Planck’s constant. The vibrational free energy (Figure 9), including zero-point eigenmodes for Si_136_ obtained by us using the HA method with the VASP code, is consistent with the results reported by Miranda et al. [42]. They determined the Gibbs free energy at zero pressure (*P* = 0), which is equivalent to the vibrational free energy *F*_vib_ because the *PV* term vanishes. Moreover, below we show such a thermodynamic feature that lies within the low-temperature regime in the temperature range from 10 K to 400 K. Our results for *F*_vib_ show that the *F*_vib_ values decrease from 0.06 eV/atom at *T* = 10 K to approximately 0.01 eV/atom at *T* = 400 K.

**Figure 10:**
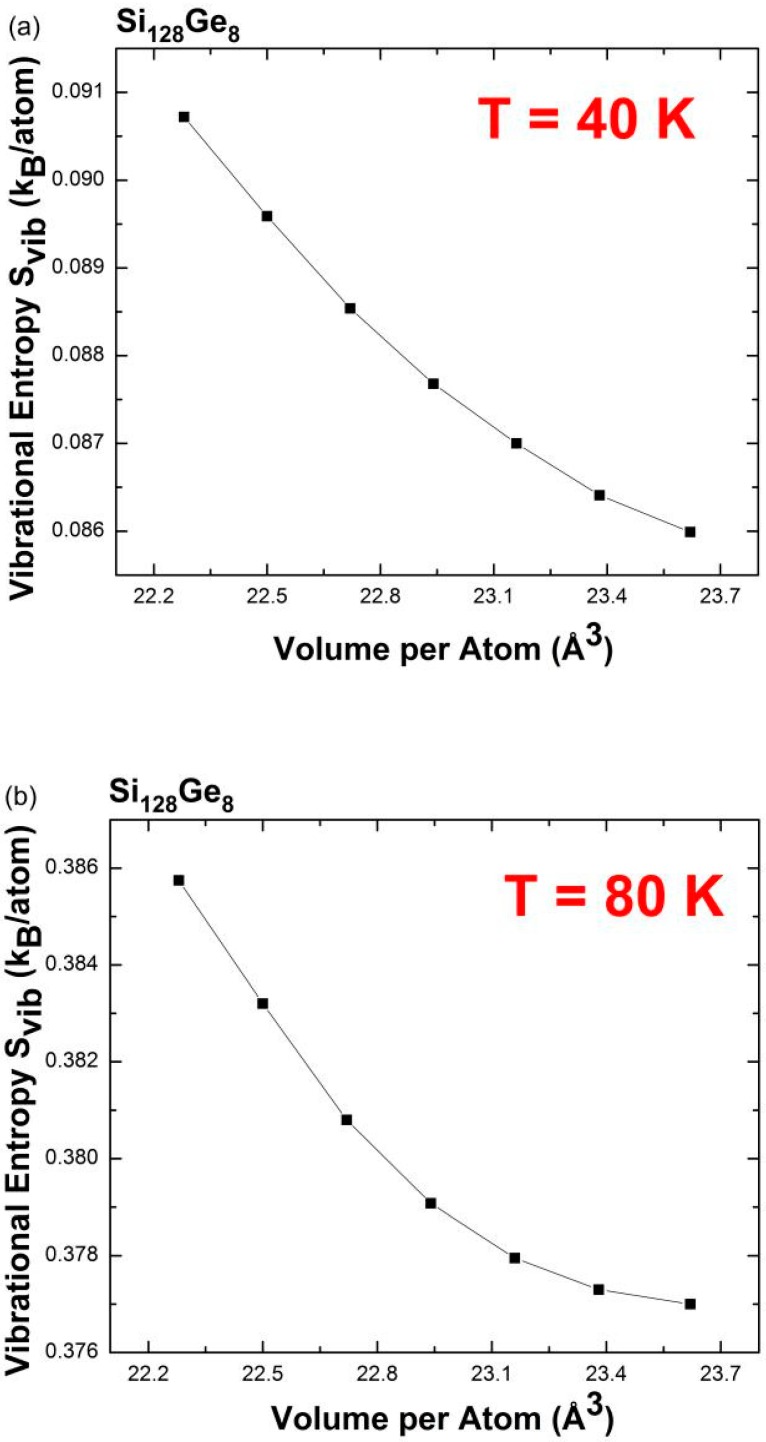

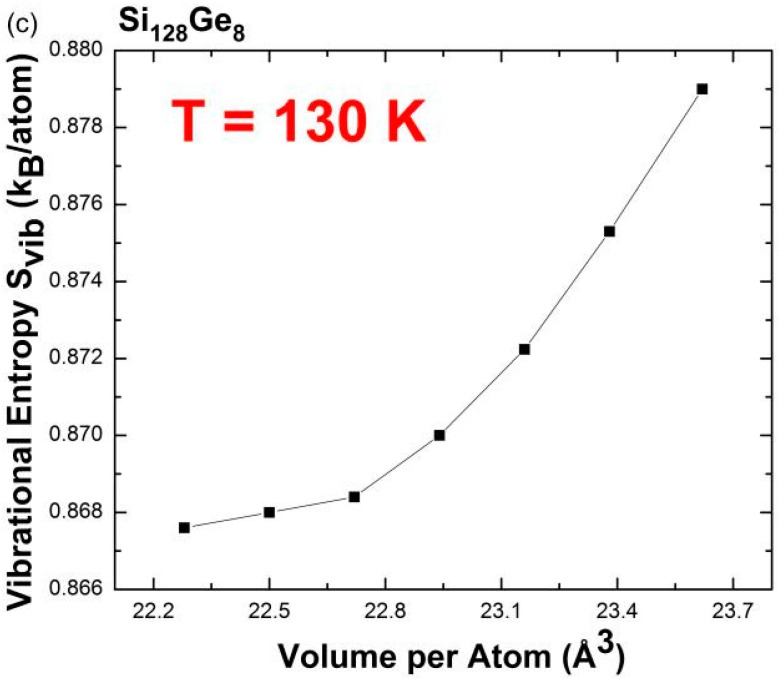
Predicted vibrational entropy as a function of the designated volume at (**a**) *T* = 40 K, (**b**) *T* = 80 K, and (**c**) *T* = 130 K for Si_128_Ge_8_.

In the same approximation, the vibrational entropy derived from the vibrational free energy at constant volume is given by *S*_vib_ = −(ə*F*_vib_/ə*T*)_V_. Based on the original definition for the volumetric thermal expansion coefficient (VCTE) *α*_V_ = *V*^−1^(ə*V*/ə*T*)_P_ and the Maxwell relationship (ə*P*/ə*T*)_V_ = (ə*S*_vib_/ə*V*)_T_, we find that the VCTE is proportional to the sums of the mode contributions to the vibrational entropy as given by *α*_V_ = (1/*B*)[ə∑**_q_**_,j_*S*_j_(*ω*_j_(***q***))/ə*V*] [43]. Here, *B* is the bulk modulus. Our first-principles calculations determine the macroscopic vibrational entropy *S*_vib_ that replaces ∑**_q_**_,j_*S*_j_(*ω*_j_(***q***)). A similar approach has been outlined in references [5,44]. It is known that the wave vector sampling of this procedure spans over 255 points within the Brillouin zone for ***q***-integration. More reliable predictions are obtained when finer ***q***-point grids are used. Based on the finite difference method, the volume derivative with respect to the vibrational entropy *S*_vib_ is approximated as Δ*S*_vib_*/*Δ*V*. Consequently, this provides an alternative way for exploring anomalous NTE through the change in the vibrational entropy (Δ*S*_vib_) with respect to the change in volume (Δ*V*) at the specified temperature. A positive Δ*S*_vib_*/*Δ*V* gives rise to a positive thermal expansion coefficient. Accordingly, we calculated S_vib_ for a small number of designated atomic volumes at several characteristic temperatures, as shown below. To better illustrate the effect of the volume change on the vibrational entropy, our plotted *S*_vib_ vs. *V* are shown using expanded unit cells that are 1%, 2%, 3% larger than the optimized geometry and the contracted unit cells that are 1%, 2%, 3% smaller than the equilibrium volume. The solid line acts as a guideline for determining the Δ*S*_vib_*/*Δ*V* ratio for each small line segment when connecting these discrete data points in a smoothly continuous manner, leading to a qualitatively reasonable estimation of VCTE. Figure 10 shows the predicted vibrational entropy of Si_128_Ge_8_, at *T* = 40 K, 80 K, 130 K in terms of volume per atom. The increasing behavior of *S*_vib_ is observed in Figure 10c, whereas *T* = 130 K is slightly smaller than the upper limit of the NTE temperature range found for Si_136_ (~140 K) [7]. The negative slopes for each small line segment appearing in Figure 10a,b are indicative of NTE behavior at *T* = 40 K and *T* = 80 K. In other words, the lattice framework of Si_128_Ge_8_ contracts upon heating from lower temperatures. 

**Figure 11:**
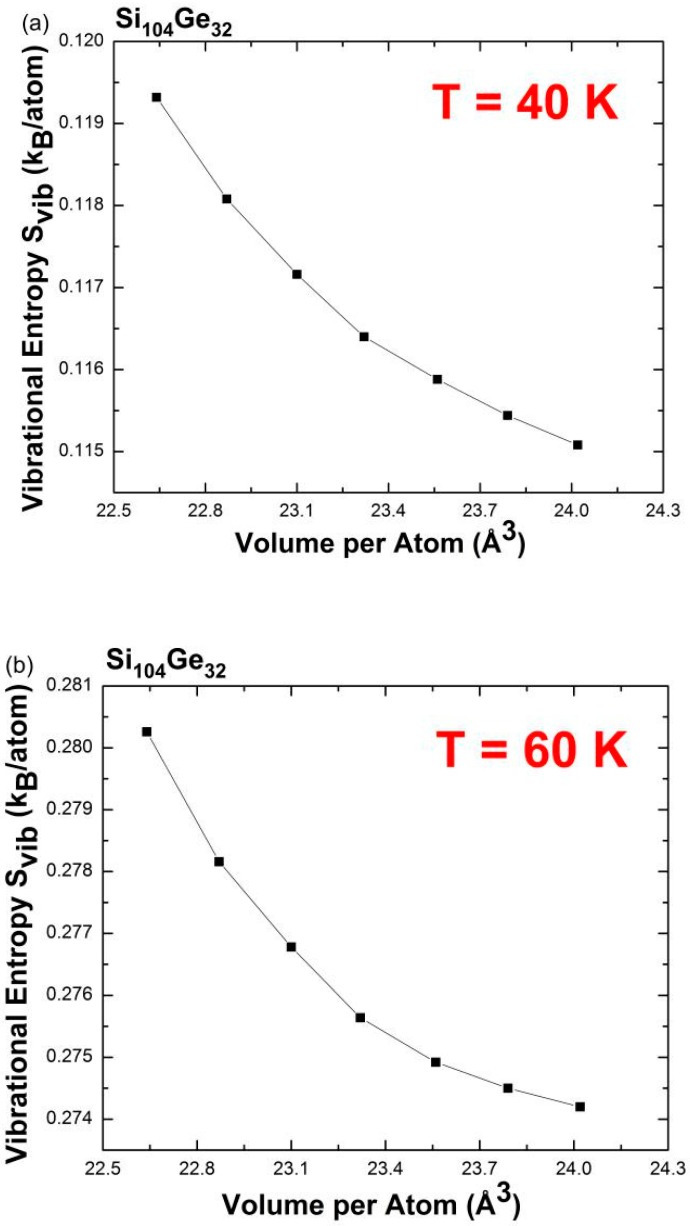

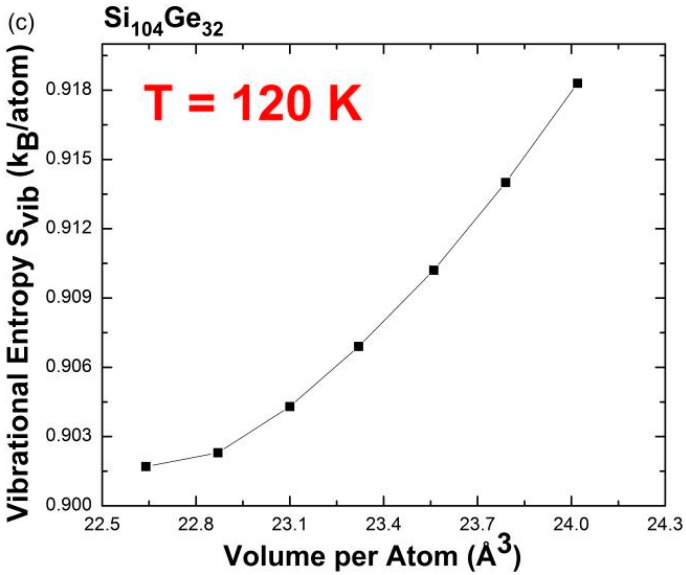
Predicted vibrational entropy as a function of the designated volume at (**a**) *T* = 40 K, (**b**) *T* = 60 K, and (**c**) *T* = 120 K for Si_104_Ge_32_.

To revisit the NTE behavior by focusing on the so-called entropy-driven effect [8], the Ge concentration *x* in Si_136-*x*_Ge*_x_* was raised from 8 to 32. Figure 11 shows a series of plots of predicted volume-dependent *S*_vib_ at different temperatures for Si_104_Ge_32._ From these continuous curves, the slopes of the vibrational entropy as a function of lattice volume for each line segment are found to be negative at *T* = 40 K and *T* = 60 K, respectively, while remaining positive at *T* = 120 K. Therefore, the NTE phenomenon is found to be present at *T* = 40 K (and *T* = 60 K) but vanishes at *T* = 120 K. It is therefore anticipated that the slopes change their sign from negative to positive at some finite temperature between *T* = 60 K and *T* = 120 K, beyond which the disappearance of the NTE phenomenon is expected. The previously predicted NTE temperature range (0—84 K) for Si_104_Ge_32_ appearing in Figure 8 indicates that the NTE effect begins to disappear at approximately 87 K which is nearly at the midpoint of the 60–120 K range displayed in Figure 11b,c. Similarly, for the Si_128_Ge_8_ alloy, quantities involving Δ*S*_vib_*/*Δ*V* switch their sign from negative to positive when the temperature is increased from 80 K to 130 K. Additionally, in Figure 8 the upper limit for the existence of NTE is approximately 108 K, similar to the average of *T* = 80 K and *T* = 130 K specified in Figure 10a,b. Finally, both of these two approaches utilizing the quasi-harmonic approximation method give rise to a substantially consistent result when predicting anomalous negative thermal expansion behavior at specified temperatures, and specify the approximate *T* range within which the NTE is present.

## 4. Conclusions

In conclusion, we theoretically investigated the electronic and vibrational properties of the Si_136-*x*_Ge*_x_* (0 ≤ *x* < 136) alloy clathrate solid solution by combining ab initio DFT lattice dynamics with QHA. The computational results for the optical band gap of Si_136-*x*_Ge*_x_* (*x* = 8, 40, 104) observed in the EDOS profiles were compared with the values obtained by other researchers using the CASTEP code instead of VASP. Furthermore, the EDOS profiles for the valence band stayed almost unchanged as the Ge composition *x* was tuned from 8 to 40 to 104. However, a somewhat apparent variation in EDOS was found in the lower region of the conduction band when the Ge content was dominant. Based on the QHA formalism, two different approaches were applied to predict the occurrence of negative thermal expansion in the 1 K < *T* < 85 K low-temperature range for Si_128_Ge_8_ and in the 1 K < *T* < 115 K range for Si_104_Ge_32._ Specifically, the NTE temperature range of the studied Si_104_Ge_32_ clathrate alloy was found to be somewhat narrower than that of Si_136_, while Si_128_Ge_8_ has a slightly smaller temperature region of NTE than Si_136_. Our predicted vibrational free energy of Si_136_ including the zero-point eigenmodes is in relatively good agreement with the reported experimental value. The generated dispersion relationships for Si_104_Ge_32_ and Si_32_Ge_104_ are quite similar for the low-frequency (<150 cm^−1^) phonon spectrum, leading to nearly the same speed of sound. The obtained Grüneisen mode parameter determined by the dependence of the mode frequency change on the volume change provides an efficient route for evaluating the thermal expansion coefficient, which may suggest NTE behavior. At temperatures below approximately 120 K, the lowest-lying phonons, namely, the transverse acoustic phonons, make a larger contribution to producing a negative macroscopic Grüneisen parameter and the subsequent NTE phenomenon than the rest of the phonons. 

## Figures and Tables

**Table 1:** Calculations of the mode Grüneisen parameters for the theoretically studied Si_136-*x*_Ge*_x_* (*x* = 8, 32).

Mode	Critical Point	Si_128_Ge_8_	Si_104_Ge_32_
TA(1)	X	−1.37	−1.23
TA(1)	Γ	−1.42	−1.29
LA	X	0.51	0.76
LA	Γ	0.94	0.90
